# Prevalence of Colorectal Neoplasia 10 or More Years After a Negative Screening Colonoscopy in 120 000 Repeated Screening Colonoscopies

**DOI:** 10.1001/jamainternmed.2022.6215

**Published:** 2023-01-17

**Authors:** Thomas Heisser, Jens Kretschmann, Bernd Hagen, Tobias Niedermaier, Michael Hoffmeister, Hermann Brenner

**Affiliations:** 1 Division of Clinical Epidemiology and Aging Research, German Cancer Research Center, Heidelberg, Germany; 2 Medical Faculty Heidelberg, University of Heidelberg, Heidelberg, Germany; 3 Central Research Institute of Ambulatory Health Care in Germany, Berlin, Germany; 4 Division of Preventive Oncology, German Cancer Research Center and National Center for Tumor Diseases, Heidelberg, Germany; 5 German Cancer Consortium, German Cancer Research Center, Heidelberg, Germany

## Abstract

**Importance:**

Screening colonoscopy to prevent and early detect colorectal cancer is recommended to be repeated in 10-year intervals, which goes along with high demands of capacities and costs. Evidence of findings at screening colonoscopies conducted 10 or more years after a negative colonoscopy result is sparse, and it remains unclear whether screening colonoscopy intervals could possibly be prolonged.

**Objective:**

To assess the prevalence of advanced colorectal neoplasms (ADNs) at least 10 years after a negative screening colonoscopy in a very large cohort of repeated screening colonoscopy participants in Germany.

**Design, Setting, and Participants:**

This registry-based cross-sectional study on screening colonoscopy findings reported to the German screening colonoscopy registry during January 2013 to December 2019 included data on screening colonoscopies that were offered to the German general population 55 years or older since 2002; virtually all screening colonoscopies among individuals covered by Statutory Health Insurance (approximately 90% of eligible adults) are reported to the national registry. A total of 120 298 repeat screening colonoscopy participants 65 years or older were identified who had a previous negative screening colonoscopy at least 10 years prior. The findings were compared with all screening colonoscopies conducted at 65 years or older during the same period (1.25 million). The data were analyzed from March to July 2022.

**Main Outcomes and Measures:**

Prevalence of colorectal cancers and ADNs (advanced adenomas and cancers).

**Results:**

Of 120 298 participants, 72 349 (60.1%) were women. Prevalence of ADN was 3.6% and 5.2% among women and men 10 years after a negative screening colonoscopy and gradually increased to 4.9% and 6.6%, respectively, among those who had a negative colonoscopy 14 years or longer prior compared with 7.1% and 11.6% among all screening colonoscopies. Sex-specific and age-specific prevalence of ADNs at repeated colonoscopies conducted 10 or more years after a negative colonoscopy were consistently at least 40% lower among women than among men, lower at younger vs older ages, and much lower than among all screening colonoscopies (standardized prevalence ratios for cancers: 0.22-0.38 among women, 0.15-0.24 among men; standardized prevalence ratios for ADNs: 0.49-0.62 among women, 0.50-0.56 among men).

**Conclusions and Relevance:**

The results of this cross-sectional study suggest that ADN prevalence at screening colonoscopies conducted 10 or more years after a negative screening colonoscopy is low. Extension of the currently recommended 10-year screening intervals may be warranted, especially for female and younger participants without gastrointestinal symptoms.

## Key Points

**Question:**

Could currently recommended 10-year screening colonoscopy intervals be extended?

**Findings:**

In this registry-based cross-sectional study, prevalences of advanced neoplasms were 40% to 50% lower among 120 098 participants who underwent repeated screening colonoscopy 10 or more years after a negative colonoscopy than among all screening colonoscopies (1.25 million). The prevalence of advanced neoplasms was at least 40% lower in women vs men, and particularly low for repeated colonoscopies conducted for those younger than 75 years.

**Meaning:**

The study results suggest that extension of the currently recommended 10-year screening intervals may be warranted, especially for female and younger participants.

## Teaser

This cross-sectional study examines the prevalence of advanced colorectal neoplasms at least 10 years after a negative screening colonoscopy in repeated screening colonoscopy participants in Germany.

## Introduction

Screening colonoscopy has been shown to reduce colorectal cancer (CRC) incidence and mortality by enabling detection and removal of precancerous lesions.^1,2,3^ However, the available evidence about the optimal screening interval is limited. A systematic review and meta-analysis of 28 studies published in 2019 found that detection of advanced neoplasms within 10 years after a negative index colonoscopy is rare.^4^ This suggests that 10-year intervals for screening colonoscopy, as currently recommended by major US and international guidelines,^5,6^ may be adequate.

However, as most of the studies identified by the review had low sample sizes (mostly limited to several hundred individuals), more studies involving larger groups of participants are needed to strengthen the empirical basis for more pertinent recommendations. In addition, uncertainty remains regarding a potential expansion of the 10-year period, as to our knowledge, evidence on longer intervals between colonoscopic examinations is very sparse. For instance, the previously mentioned review identified only 2 studies^7,8^ that reported outcomes at a follow-up colonoscopy for participants who had an interval of at least 10 years. Similarly, most of the available studies did not stratify by sex and age, 2 main CRC risk factors.^9,10^ Evidence on potential differences by sex or age could help to establish risk-adapted screening strategies, eg, by using longer intervals or less invasive tests for those at lower risk and offering more intensive screening for participants at increased risk. More targeted screening offers would potentially reduce the burden of testing and demand of capacities and costs associated with colonoscopy,^11^ thereby also counteracting the frequently reported overuse and underuse of screening examinations in considerable proportions of the population.^12,13,14,15^

To strengthen the evidence base of current recommendations, as well as to generate more insights on a potential prolongation of colonoscopy screening intervals and the possible benefit of risk stratification, we assessed the prevalence of colorectal neoplasms at least 10 years after a negative index colonoscopy as stratified by interval, sex, and age in a very large cohort of repeated screening colonoscopy participants in Germany.

## Methods

### Data Sources

This analysis was based on data of the German screening colonoscopy registry, the world’s largest registry of its kind.^16,17^ Anonymized registration of screening colonoscopy findings and the use of the anonymized data for program evaluation by the Central Research Institute of Ambulatory Health Care in Germany is mandatory in the German screening colonoscopy program. Only aggregated data from the screening colonoscopy registry (which includes registration of approximately 10 million screening colonoscopies) provided by this institution were used for this analysis. Primary screening colonoscopy for prevention and early detection of CRC has been offered in Germany since October 2002. Eligibility starts at age 50 years for men (lowered from age 55 years only in April 2019) and age 55 years for women. If this first screening colonoscopy is conducted before age 65 years, a second screening colonoscopy is offered 10 years later. In Germany, certification for conducting screening colonoscopies is tightly regulated based on extensive previous training and experience, and its maintenance is subject to rigorous quality control. Specifically, only gastroenterologists, internists, or surgeons who have conducted at least 200 colonoscopies during the preceding 2 years will be certified. Maintenance of certification is contingent on conducting at least 200 colonoscopies per year, the quality and completeness of which needs to be proven by photo or video documentation.

Along with introducing the screening colonoscopy offer, a national registry was built that included all screening colonoscopies among individuals covered by Statutory Health Insurance (approximately 90% of eligible adults). Further details on the registry have been provided elsewhere.^16,17^ All eligible screening colonoscopies are reported anonymously on a standardized form. Colonoscopies for the clarification of symptoms or after a positive stool test, which is also offered in Germany, are not included. Reporting is virtually complete, as it is a prerequisite for physicians’ reimbursement by the health insurance funds. Reported items include identifiers for the medical practice and patient (as assigned by the medical practice); basic sociodemographic variables, such as sex and age; and information on findings at colonoscopy, including number, size, and histologic characteristics of polyps. In cases of multiple neoplasms, only characteristics of the most advanced type are recorded.

### Study Population

Due to anonymized recording of screening colonoscopies in the registry, direct identification of all repeated colonoscopies was not possible. However, through an algorithm detailed in the eMethods in Supplement 1, a subgroup with repeated colonoscopies conducted at 65 years or older during the years 2013 to 2019 that were conducted 10 or more years after an initial negative screening colonoscopy could be reliably identified. The repeated screening colonoscopies were compared with all screening colonoscopies conducted at 65 years or older from 2013 to 2019, most of which were first screening colonoscopies. The comparison group of all screening colonoscopies also included an unknown, but likely small, fraction of patients with repeated screening colonoscopy use (including, but not limited to, the subset of individuals reliably identified by the algorithm mentioned previously). As lower prevalence in repeated screening participants is expected, use of the entire data set constitutes a conservative approach for comparison.

### Statistical Analysis

Given the expected differences in adenoma prevalence by sex, all analyses were conducted separately for men and women. We first described the population characteristics and then assessed the prevalence of any neoplasm (any adenoma or cancer [ANN]), any advanced neoplasm (advanced adenoma or cancer [ADN]) and CRC in those with 1 prior screening colonoscopy without a neoplastic finding compared with all participants with screening colonoscopy. In the German screening colonoscopy registry, advanced adenomas are defined as at least 1 adenoma greater than 1 cm or at least 1 adenoma with villous components or high-grade dysplasia. Further stratification was made by interval since negative index colonoscopy. Prevalence was assessed overall as well as stratified by age (65-69, 70-74, 75-79, and ≥80 years). For repeated colonoscopy users, we used the age at the second screening colonoscopy. To evaluate for a trend with increasing length of follow-up, χ^2^ tests for trend in proportions were performed.

Lastly, we compared the observed numbers of cases with CRCs and ADNs in repeated screening colonoscopy users with the numbers of cases expected if the same sex-specific and age-specific prevalence rates were observed in this group as in all screening colonoscopy participants. Standardized prevalence ratios (SPRs) stratified by sex and age (65-69, 70-74, ≥75 years) were calculated as the ratio of observed and expected numbers, along with exact 95% CIs.^18^ All analyses were conducted in R, version 4.0.2 (R Foundation), and statistical significance was set at an α of 5%.

## Results

### Population Characteristics

Table 1 shows main characteristics of the study population. We identified 47 949 men (39.9%) and 72 349 women (60.1%) whose colonoscopy could be reliably classified as repeated screening colonoscopy of 565 864 and 688 264 screening colonoscopies in men and women 65 years or older, respectively, that were recorded in the German screening colonoscopy registry from January 1, 2013, to December 31, 2019.

**Table 1.** Characteristics of the Study Population

Characteristic	2013-2019, No. (%)	
	Repeated screening colonoscopy users	All screening colonoscopy users
Sex		
Men	47 949 (39.9)	565 864 (45.1)
Women	72 349 (60.1)	688 264 (54.9)
Age at (last) screening colonoscopy, y		
65-69	49 160 (40.9)	511 708 (40.8)
70-74	40 322 (33.5)	388 840 (31.0)
75-79	24 504 (20.4)	265 127 (21.1)
≥80	6312 (5.2)	88 453 (7.1)
Most advanced finding at previous screening colonoscopy		
Nonneoplastic finding	34 741 (28.9)	NA
No finding	85 557 (71.1)	
Years between first and second screening colonoscopy		
10	58 978 (49.0)	NA
11	34 762 (28.9)	
12	14 427 (12.0)	
13	6786 (5.6)	
14	3374 (2.8)	
15	1522 (1.3)	
16	449 (0.4)	

Abbreviation: NA, not applicable.

Compared with all individuals screened, repeated screening colonoscopy users were more likely to be female (60.1% vs 54.9%). The age structure was overall comparable between the groups. Among repeated screening colonoscopy users, most (85 557 [71.1%]) had no finding at the previous screening colonoscopy, and 34 741 (28.9%) had nonneoplastic findings, such as hyperplastic polyps. Approximately half of individuals (58 978 [49.0%]) used a second screening colonoscopy 10 years after the preceding negative screening colonoscopy. Of the remainder, 34 762 (28.9%) and 14 427 (12.0%) used repeated screening 11 years and 12 years later, respectively, and only approximately 10% had a screening interval of 13 or more years.

### Overall Prevalence

**Figure 1.**
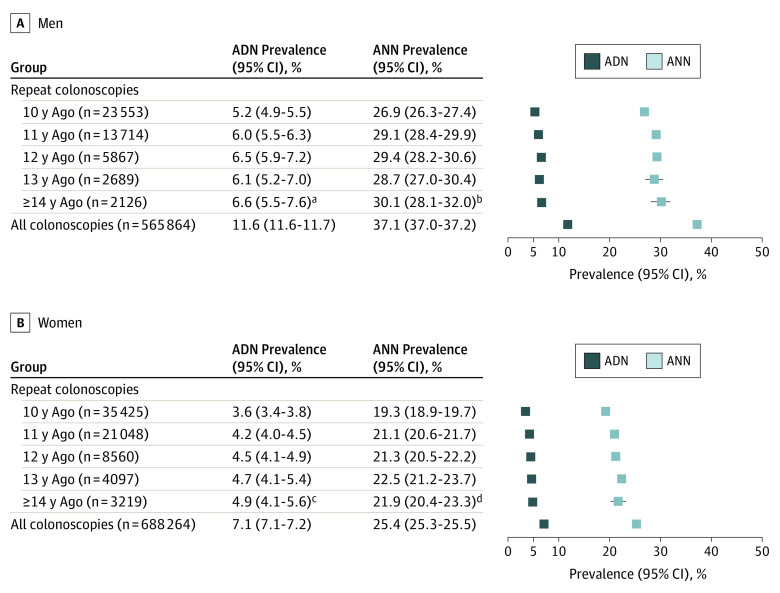
Findings at Screening Colonoscopy During 2013 to 2019 in Repeated Screening Participants and Overall as Stratified by Individual Years Since Previous Colonoscopy χ^2^ Test for trend in proportions. ADN indicates prevalence of any advanced neoplasm; ANN, prevalence of any neoplasm. ^a^χ^2^ = 19.20; *P* < .001. ^b^χ^2^ = 23.40; *P* < .001. ^c^χ^2^ = 33.31; *P* < .001. ^d^χ^2^ = 42.17; *P* < .001.

Figure 1 shows the ANN and ADN prevalence as stratified by sex and, for repeated screening colonoscopy users, interval since the last negative colonoscopy. Among repeated screening colonoscopy users, prevalence gradually increased with time intervals since negative index colonoscopy (from 5.2% to 6.6% in men and 3.6% to 4.9% in women for intervals of 10, 11, 12, 13, and ≥14 years). Even the prevalence 14 years or greater after a negative screening colonoscopy was markedly lower than those in all screening colonoscopy users, for whom ADNs were found in 65 911 men (11.6%) and 49 100 women (7.1%).

**Figure 2.**
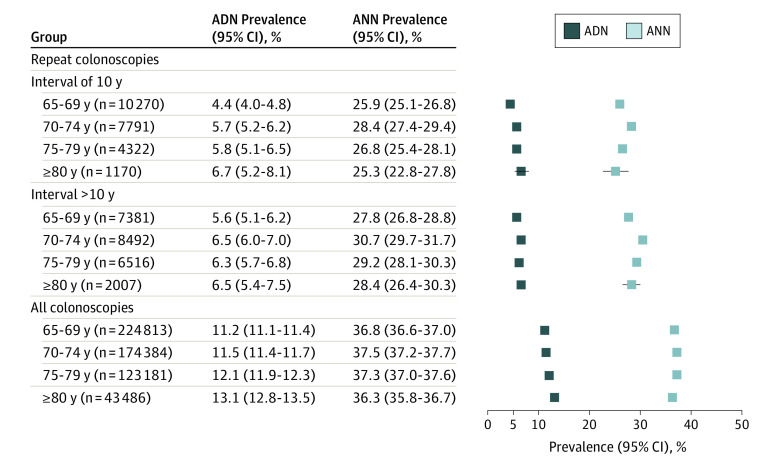
Findings at Screening Colonoscopy From 2013 to 2019 in Repeated Screening Participants and Overall as Stratified by Age for Men ADN indicates prevalence of any advanced neoplasm; ANN, prevalence of any neoplasm.

**Figure 3.**
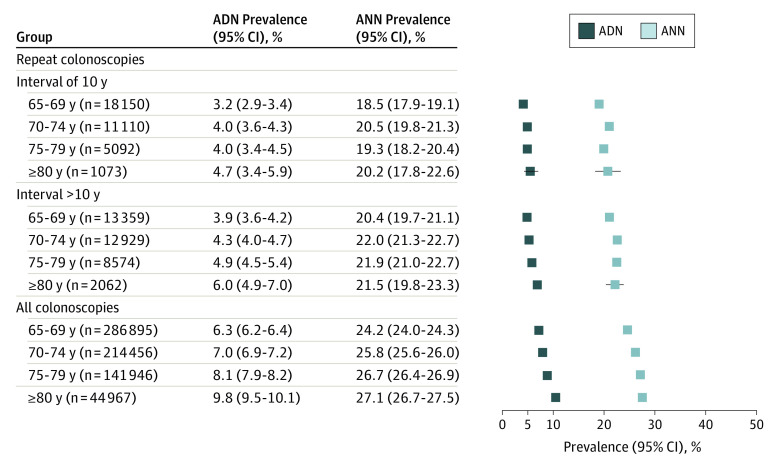
Findings at Screening Colonoscopy From 2013 to 2019 in Repeated Screening Participants and Overall as Stratified by Age for Women ADN indicates prevalence of any advanced neoplasm; ANN, prevalence of any neoplasm.

Further stratification by age revealed a modest trajectory toward higher ADN prevalence, but not ANN prevalence, with older ages overall and in repeated screening colonoscopy users (Figure 2 and Figure 3; see also eFigure in Supplement 1 for prevalence of advanced adenomas and cancers by 1-year intervals since negative index colonoscopy). The total detection rates of cancers (regardless of interval) ranged from 0.2% to 0.9% in repeated screening colonoscopy users and 0.6% to 3.0% in all screening colonoscopy users, with the highest detection rates among individuals 75 years or older.

### Prevalence Ratios

Observed numbers of cancers and ADNs in participants with previous negative colonoscopy were significantly lower than those expected based on the sex-specific and age-specific prevalence seen in all screening colonoscopy participants (Table 2). The corresponding SPRs ranged from 0.15 to 0.38 for cancers and 0.40 to 0.62 for ADN and tended to be slightly higher in women vs men and for screening intervals longer than 10 years vs 10 years. However, even in women with an interval that was longer than 10 years, the (standardized) prevalence of cancers and ADN was 62% to 74% and 38% to 39% lower, respectively, after a previous negative colonoscopy compared with overall screening colonoscopy participants.

**Table 2.** Observed and Expected Numbers and Standardized Prevalence Ratios of Colorectal Cancer and Any Advanced Neoplasms in Repeated Screening Colonoscopy Participants

Interval between screening colonoscopies and age at colonoscopy^a^	Men			Women		
	Observed, No. (%)	Expected, No.^b^	SPR (95% CI)	Observed, No. (%)	Expected, No.^b^	SPR (95% CI)
**Cancer**						
Interval of 10 y						
≥65 y	73 (0.3)	373.3	0.20 (0.15-0.25)	66 (0.2)	289.7	0.23 (0.18-0.29)
65-69 y	19 (0.2)	129.7	0.15 (0.09-0.23)	27 (0.1)	109.3	0.25 (0.16-0.36)
70-74 y	25 (0.3)	120.5	0.21 (0.13-0.31)	20 (0.2)	93.0	0.22 (0.13-0.33)
≥75 y	29 (0.5)	123.1	0.24 (0.16-0.34)	19 (0.3)	87.4	0.22 (0.13-0.34)
Interval >10 y						
≥65 y	87 (0.4)	417.3	0.21 (0.17-0.26)	118 (0.3)	341.6	0.35 (0.29-0.41)
65-69 y	21 (0.3)	93.2	0.23 (0.14-0.34)	21 (0.2)	80.5	0.26 (0.16-0.40)
70-74 y	24 (0.3)	131.4	0.18 (0.12-0.27)	41 (0.3)	108.2	0.38 (0.27-0.51)
≥75 y	42 (0.5)	192.7	0.22 (0.16-0.29)	56 (0.5)	152.9	0.37 (0.28-0.48)
**Any advanced neoplasm**						
Interval of 10 y						
≥65 y	1226 (5.2)	2725.9	0.45 (0.42-0.48)	1269 (3.6)	2446.7	0.52 (0.49-0.55)
65-69 y	457 (4.4)	1153.6	0.40 (0.36-0.43)	578 (3.2)	1149.5	0.50 (0.46-0.55)
70-74 y	441 (5.7)	896.5	0.49 (0.45-0.54)	439 (4.0)	782.0	0.56 (0.51-0.62)
≥75 y	328 (6.0)	675.8	0.49 (0.43-0.54)	252 (4.1)	515.2	0.49 (0.43-0.55)
Interval >10 y						
≥65 y	1505 (6.2)	2857.1	0.53 (0.50-0.55)	1630 (4.4)	2648.7	0.62 (0.59-0.65)
65-69 y	415 (5.6)	829.2	0.50 (0.45-0.55)	521 (3.9)	846.1	0.62 (0.56-0.67)
70-74 y	552 (6.5)	977.2	0.56 (0.52-0.61)	563 (4.4)	910.1	0.62 (0.57-0.67)
≥75 y	538 (6.3)	1050.7	0.51 (0.47-0.56)	546 (5.1)	892.5	0.61 (0.56-0.67)

Abbreviation: SPR, standardized prevalence ratio.

^a^ Age at repeated screening colonoscopy.

^b^ Expected based on the sex-specific and age-specific prevalence observed among 1 254 128 screening colonoscopy participants from 2013 to 2019.

## Discussion

Screening colonoscopy has been shown to reduce CRC incidence,^1,2,3^ but implementation with screening at currently recommended 10-year intervals may surpass colonoscopy capacities in many countries. In this study, we assessed the sex-specific and age-specific prevalence of colorectal neoplasms in a very large cohort of repeated screening colonoscopy participants (n > 120 000). The large sample size allowed us to obtain highly precise prevalence. Compared with all screening colonoscopies in the observation period, we found that the prevalence of CRC and ADN was 75% to 85% and 44% to 60% lower, respectively, 10 years after a negative colonoscopy and still 62% to 82% and 38% to 50% lower, respectively, with intervals from 11 to 16 years. Consistent with findings for all screening colonoscopies, the prevalence of ADN was substantially (approximately 40%) higher in men vs women using a repeated screening colonoscopy regardless of the interval between examinations. Among women younger than 75 years at repeated screening, prevalence of ADN was very low (approximately 4%, including <0.5% cancers) even at intervals of up to 13 years, and only slightly higher (4%-6%) at intervals 14 years or greater.

### Findings in Context

This cross-sectional study potentially adds several important aspects to the literature. First, this study underpins and expands previous evidence showing that detection of advanced findings up to 10 years after a negative index colonoscopy is rare.^4,19^ The consistently low yield of advanced findings within 10 years across this and previous studies provides reassuring evidence that screening colonoscopy need not be repeated earlier than 10 years, as currently recommended but nevertheless frequently done in clinical practice.^12,13,14,15^

Second, this study provides prevalence estimates for intervals longer than 10 years at a high level of precision. To our knowledge, only 2 small previous studies reported ADN prevalence for participants who had an interval of more than 10 years between colonoscopic examinations. The first was a cross-sectional study from Germany, which found ADN prevalence of 5.4%, 4.7%, and 7.4% for intervals of 11 to 15 years (study population, 483 participants), 16 to 20 years (n = 215), and greater than 20 years (n = 270), respectively.^19^ The second was a database review of outpatient screening colonoscopies in a private clinic in Atlanta, Georgia, which reported an 8% ADN prevalence for an interval of 10 to 15 years after negative colonoscopy (n = 363).^8^ With advanced findings of 4.2% to 6.6% (including <0.8% cancers) more than 10 years after a negative index colonoscopy (based on 61 320 participants), our findings constitute evidence that ADN prevalence is still low even more than 10 years after a negative colonoscopy. This is further supported by the available literature on cohort^20,21,22,23,24^ and case-control^25,26^ studies that exclusively focused on the rate of cancers, suggesting low CRC risks up to 20 years after a negative colonoscopy.

Third, the present study adds to the evidence on differences by sex and age in repeated screening colonoscopy participants. Male sex is well known to be associated with higher ADN and CRC risks (based on biological as well as behavior factors),^10,27,28^ as also evidenced in the approximately 60% higher ADN prevalence in men vs women in the control group. While such varying CRC risks may already support some sex-specific screening recommendations (such as varying initiation ages for screening colonoscopy in Germany),^29^ in the repeated screening setting, the available evidence did not allow us to draw conclusions regarding the consistency and magnitude of potential differences by sex. Previously reported ADN prevalence ranged from 0.7% to 7.2% for men and 0.6% to 4.6% for women within 10 years, and from 6.0% to 8.3% for men and 4.5% to 6.7% for women with intervals of more than 10 years.^4,8,19^

This study showed a pattern of significantly higher ADN prevalence in men vs women using repeated screening colonoscopy. These sex-related differences were present regardless of the interval between examinations and were most pronounced for those with repeated colonoscopies between age 65 and 74 years. The ADN prevalence was particularly low (approximately 4%, including <0.5% cancers) in women younger than 75 years even at intervals of up to 13 years. Conversely, while still considerably lower compared with first-time screening use, ADN prevalence in men at the same age and colonoscopy intervals were at least 40% higher than in women, typically in a range of 6% to 7%. Due to the large sample size, these prevalence estimates come with narrow confidence bands: the upper boundary of the 95% CI for the ADN prevalence in women younger than 75 years with intervals greater than 10 years was less than 5%, which would still be significantly lower than the ADN prevalence in first-time screened women of the same age.

The study findings also suggest that age may be more strongly associated with higher prevalence at repeated screening than the interval between examinations. Within the same sex, prevalence of advanced findings in those aged 65 to 74 years with intervals of 13 years or longer was on the same level and partly even lower than those seen in individuals 75 years or older with 10-year intervals. This may not be unexpected, as older age is generally a risk factor for CRC.^9^ However, it has largely been unknown whether and to what extent these well-described increases in CRC risks along an age trajectory would also manifest in those free of polyps at an index colonoscopy, and the limited previous evidence in this respect has been inconclusive.^19,30^

Taken together, the strong and consistent differences between men and women, as well as the lower prevalence among younger vs older repeated screening participants, indicates the potential use of risk stratification by sex and age in defining screening colonoscopy intervals. For instance, women at younger screening ages with no finding at an index colonoscopy could possibly be screened at prolonged intervals or, alternatively, be offered less invasive methods, such as stool tests, while maintaining the 10-year interval for men and women at older ages. By reducing the burden of testing in those at lower risk, such risk-adapted intervals could promote a more efficient use of colonoscopy capacities and resources regardless of a country’s income level.^31^ Future studies should assess the potential implications of risk-adapted intervals from a health-economic point of view. Possibly, cost-effectiveness analyses on CRC screening may need to be carefully revised to reflect that participants free of polyps at an index colonoscopy have a long-lasting lower risk of advanced colorectal neoplasia.

In the present study, instead of primarily comparing prevalence at repeated screening across varying interval lengths (eg, 10 vs ≥10 years), we deliberately compared prevalence at repeated screening with those at all screening colonoscopies during the same period. This approach was chosen as the detection rate at first-time screening is a benchmark for introducing and maintaining population-based screening colonoscopy programs, such as in the US or in Germany. Considering increasing colonoscopy demand due to demographic aging against the backdrop of ever-growing resource constraints in the health care sector in many countries,^32^ it appears plausible if not imperative to also evaluate repeated screening outcomes against this benchmark, which may also guide determining the clinical relevance of the prevalence seen at repeated screening on a population level. In this context, the perception of clinical relevance may vary: no clear-cut criterion exists as to what proportion of advanced neoplasms are eventually acceptable, and, on a patient-individual level, different levels of risk tolerance may be associated with different decisions. Therefore, when advising patients, health care professionals and physicians may consider communicating this study’s findings in an open and transparent manner.

Finally, this study specifically focuses on asymptomatic individuals at average risk of CRC with negative index screening colonoscopy who opted to undergo a repeated screening colonoscopy 10 or more years later. Therefore, broader generalizing of this study’s findings, which do not extend to individuals who might need to undergo a colonoscopy for clarification of symptoms (eg, rectal bleeding) at earlier intervals or individuals at higher risk of CRC (eg, inflammatory bowel disease), should be done cautiously.

### Limitations

Due to anonymization of records at the screening colonoscopy registry, we were not able to include all repeated screening colonoscopies during the observation period, as repeated use was not a documented item in the German colonoscopy registry until 2019. As a workaround, we defined an algorithm designed to identify individuals who had used a repeated screening colonoscopy. Specifically, apart from matching interval, sex, and age, a given patient must have had their examinations in the same medical practice and the same patient identifier for both colonoscopies. Theoretically, a medical practice may have reassigned a patient identifier to a new patient. Therefore, although it cannot be excluded that very few patients may not have had a repeated examination, it appears highly unlikely that such a new patient with a reassigned patient identifier would also match all the other criteria for interval, sex, and age.

Furthermore, the possibility of a healthy participants bias in those using repeated screening cannot be ruled out, as these participants might be even more health conscious than the group of individuals using first-time screening but not following up with a repeated screening colonoscopy (for whom no data are available). However, such differences within the group of screening users are probably very small. Also, some participants of repeated screening colonoscopy might have had a colonoscopy for diagnostic purposes between their first and second screening colonoscopy, which would not have been recorded in the screening colonoscopy registry. However, we would expect the numbers of such participants to be very small, as they would usually not be advised to have another screening colonoscopy for another 10 years after a negative diagnostic colonoscopy.

This study is further limited in that the screening colonoscopy registry did not include sufficient details to assess prevalence as stratified by proximal and distal colon, which would be of interest given the possibly varying potential for prevention. In particular, the proportion of cancers arising in serrated lesions is assumed to be higher in the proximal colon than in the distal colon and rectum,^33^ and the share of proximal colon cancer among all CRCs is higher in women than men, particularly at older ages.^34^

Moreover, German data privacy regulations impede linkage with cancer registry and mortality data to inform the association of screening colonoscopy with the prevention of CRC incidence and mortality. This lack of a follow-up also implies that interval cancers occurring before repeated colonoscopies could not be considered. However, preceding CRC diagnoses were excluded from repeated and all screening colonoscopies that were used to calculate observed and expected CRCs; therefore, they are unlikely to have been associated with major bias of the SDR estimates. Furthermore, it is reassuring that the SDR estimates for CRC prevalence at repeated colonoscopy were highly consistent with estimates of approximately 70% to 80% lower CRC incidence after a negative screening colonoscopy than in the general population.^20,21,22,23,24,25,26^ As well, missed detection (rather than biological factors [ie, the natural history]) has previously been proposed as a key driver for the occurrence of interval cancers after a negative colonoscopy.^35^ Therefore, it should be considered that, although this study supports asymptomatic, average-risk individuals to fully adhere to the recommended 10-year interval and possibly even to extend intervals for those at particularly low risk, a high procedural quality of the index colonoscopy remains an essential prerequisite.

## Conclusions

In this large cross-sectional study of repeated screening colonoscopy participants, we found a sustained low prevalence of ADN of approximately 6% to 7% in men and 4% to 5% in women even longer than 10 years after a negative colonoscopy. The prevalence of advanced neoplasms was consistently at least 40% higher in men vs women, regardless of the interval between examinations, and the sex difference was most pronounced at younger ages. The study results provide evidence that, for asymptomatic patients with a negative baseline examination, the currently recommended screening colonoscopy intervals are safe and suggest that sex and age could guide potential risk-adapted extension of screening intervals beyond 10 years, especially for female and younger participants.

## Notes

Supplement 1. eMethods. eFigure. Findings at Screening Colonoscopy in 2013-2019, in Repeat Screening Participants and Overall, Stratified by Age, Sex and Intervals

Supplement 2. Data sharing statement
