# Supplementary material for: Prevalence of Colorectal Neoplasia 10 or More Years After a Negative Screening Colonoscopy in 120 000 Repeated Screening Colonoscopies
Source: JAMA Intern Med. 2023 Jan 17;183(3):183–90. doi: 10.1001/jamainternmed.2022.6215 (PMC9857826; doi:10.1001/jamainternmed.2022.6215)
Supplement: Supplement 1. — eMethods. eFigure. Findings at Screening Colonoscopy in 2013-2019, in Repeat Screening Participants and Overall, Stratified by Age, Sex and Intervals [file jamainternmed-e226215-s001.pdf]

# Supplemental Online Content

Heisser T, Kretschmann J, Hagen B, Niedermaier T, Hoffmeister M, Brenner H. Prevalence of colorectal neoplasia 10 or more years after a negative screening colonoscopy in 120 000 repeated screening colonoscopies. *JAMA Intern Med*. Published online January 17, 2023. doi:10.1001/jamainternmed.2022.6215

**eMethods.**

**eFigure.** Findings at Screening Colonoscopy in 2013-2019, in Repeat Screening Participants and Overall, Stratified by Age, Sex and Intervals

This supplemental material has been provided by the authors to give readers additional information about their work.

© 2023 Heisser T et al. *JAMA Internal Medicine.*

## eMethods

### Algorithm to identify repeat screening colonoscopy users

Repeated screening colonoscopy use was not documented in the registry until 2019, and the overall fraction of patients with repeated colonoscopy thus remains unknown. As it was therefore not possible to include the totality of individuals with repeated use of screening colonoscopy, we identified a subset of repeat screening colonoscopy users by applying the following set of criteria designed to ensure that included examinations were indeed repeated screening colonoscopies: (1) identical medical practice and assigned patient identifier (which is only unique within the same medical practice) for both screening colonoscopies, (2) interval of at least 10 years since the first screening colonoscopy, (3) same sex, and (4) age and time interval matching with age at first screening colonoscopy.

As screening colonoscopy is offered maximally twice, documented screening colonoscopies with more than two combinations of medical practice and patient identifier were excluded. We also excluded combinations where the interval between both colonoscopies was lower than 10 years, as well as individuals with neoplastic findings at the index colonoscopy, as in both circumstances the second colonoscopy was likely a misclassified surveillance colonoscopy. The analysis was limited to the years 2013-2019, as 2013 was the first full calendar year capturing individuals undergoing repeat screening colonoscopies after the recommended and offered interval of ten years (since the offer was introduced in October 2002), and 2019 was the final year where data were available.

For comparison, we used the data of all documented screening colonoscopies for the years 2013-2019. As these also include an unknown, but likely small, fraction of patients with repeat screening colonoscopy use (including, but not limited to, the subset of individuals with very high likelihood as identified by the matching criteria described above), use of this dataset constitutes a conservative approach for comparison. As eligibility started at age 55 during the observation period and as a second screening colonoscopy is offered 10 years after an index colonoscopy without finding (i.e., at age 65), both the group of repeat colonoscopy users and the comparison group were limited to individuals aged 65 or older.

© 2023 Heisser T et al. *JAMA Internal Medicine.*

A. Ages 65-69 B. Ages 70-74

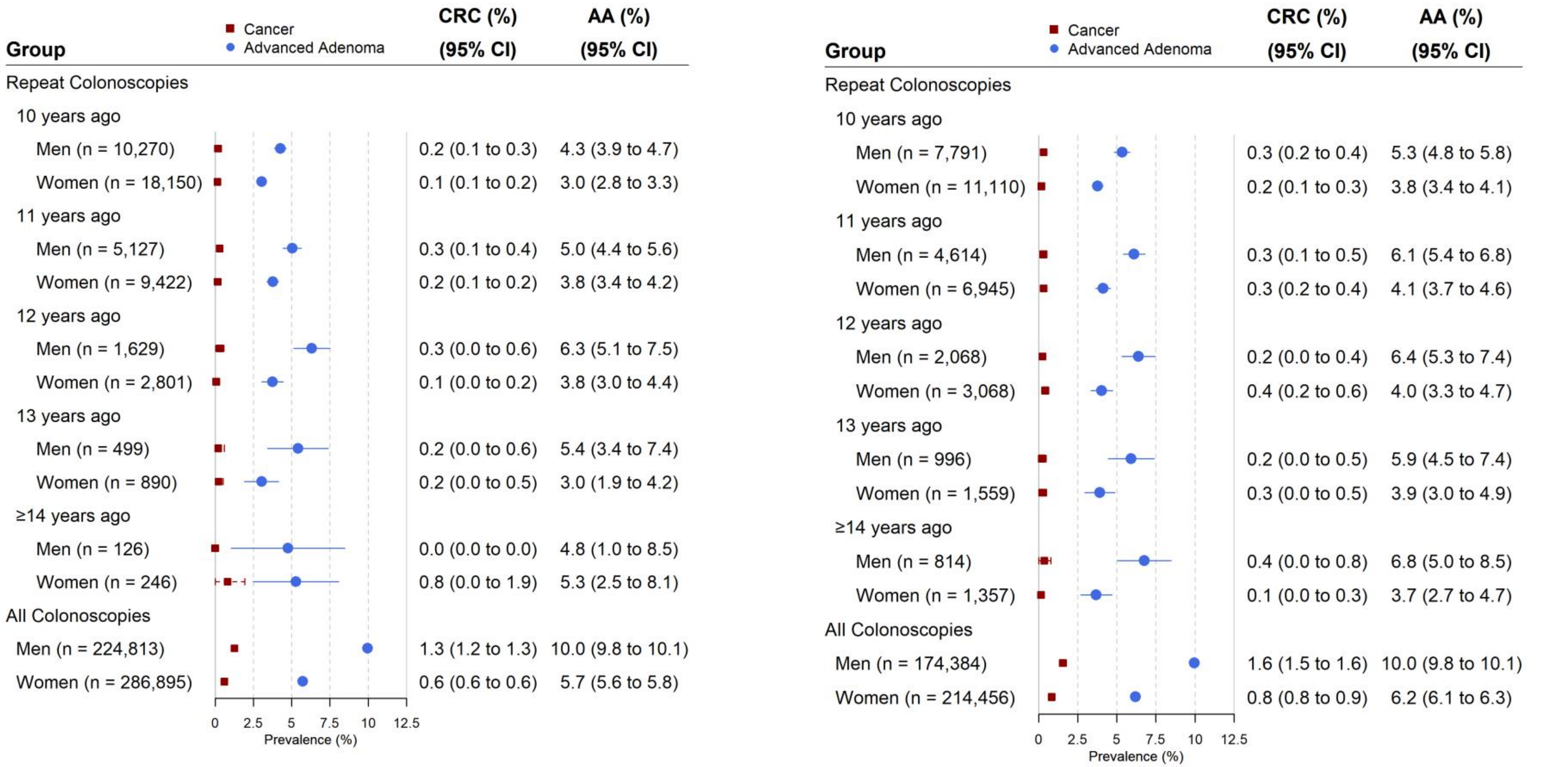


**eFigure. Findings at Screening Colonoscopy in 2013-2019, in Repeat Screening Participants and Overall, Stratified by Age, Sex and Intervals**

ADN, prevalence of any advanced neoplasm; ANN, prevalence of any neoplasm; CI, confidence interval.

© 2023 Heisser T et al. *JAMA Internal Medicine.*

C. Ages 75-79

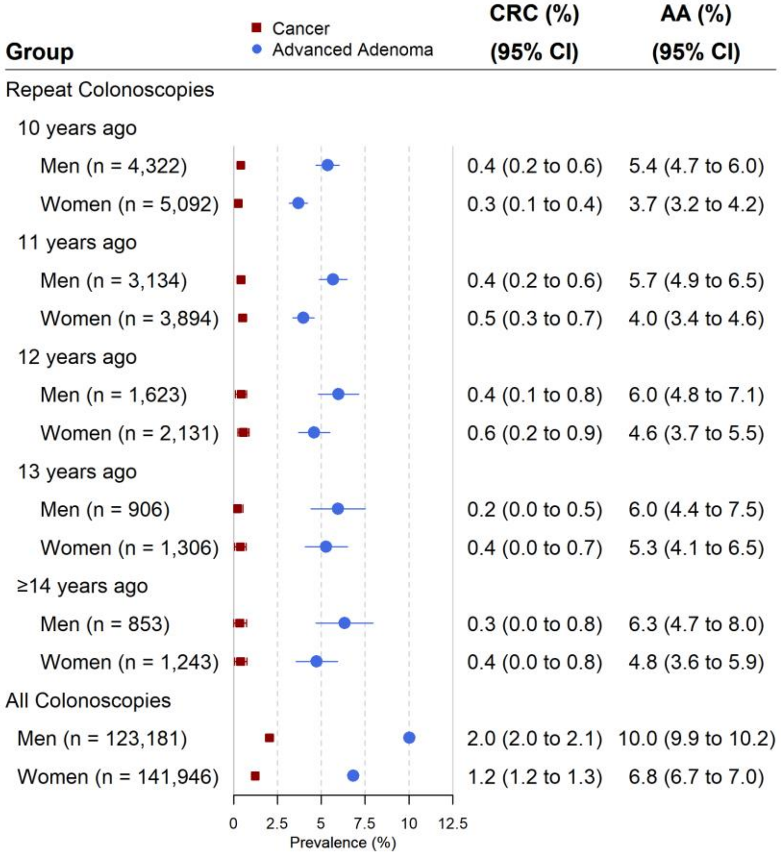


| Group | CRC (%) (95% CI) | AA (%) (95% CI) |
|---|---|---|
| Repeat Colonoscopies | | |
| 10 years ago | | |
| Men (n = 4,322) | 0.4 (0.2 to 0.6) | 5.4 (4.7 to 6.0) |
| Women (n = 5,092) | 0.3 (0.1 to 0.4) | 3.7 (3.2 to 4.2) |
| 11 years ago | | |
| Men (n = 3,134) | 0.4 (0.2 to 0.6) | 5.7 (4.9 to 6.5) |
| Women (n = 3,894) | 0.5 (0.3 to 0.7) | 4.0 (3.4 to 4.6) |
| 12 years ago | | |
| Men (n = 1,623) | 0.4 (0.1 to 0.8) | 6.0 (4.8 to 7.1) |
| Women (n = 2,131) | 0.6 (0.2 to 0.9) | 4.6 (3.7 to 5.5) |
| 13 years ago | | |
| Men (n = 906) | 0.2 (0.0 to 0.5) | 6.0 (4.4 to 7.5) |
| Women (n = 1,306) | 0.4 (0.0 to 0.7) | 5.3 (4.1 to 6.5) |
| ≥14 years ago | | |
| Men (n = 853) | 0.3 (0.0 to 0.8) | 6.3 (4.7 to 8.0) |
| Women (n = 1,243) | 0.4 (0.0 to 0.8) | 4.8 (3.6 to 5.9) |
| All Colonoscopies | | |
| Men (n = 123,181) | 2.0 (2.0 to 2.1) | 10.0 (9.9 to 10.2) |
| Women (n = 141,946) | 1.2 (1.2 to 1.3) | 6.8 (6.7 to 7.0) |

B. Ages 80+

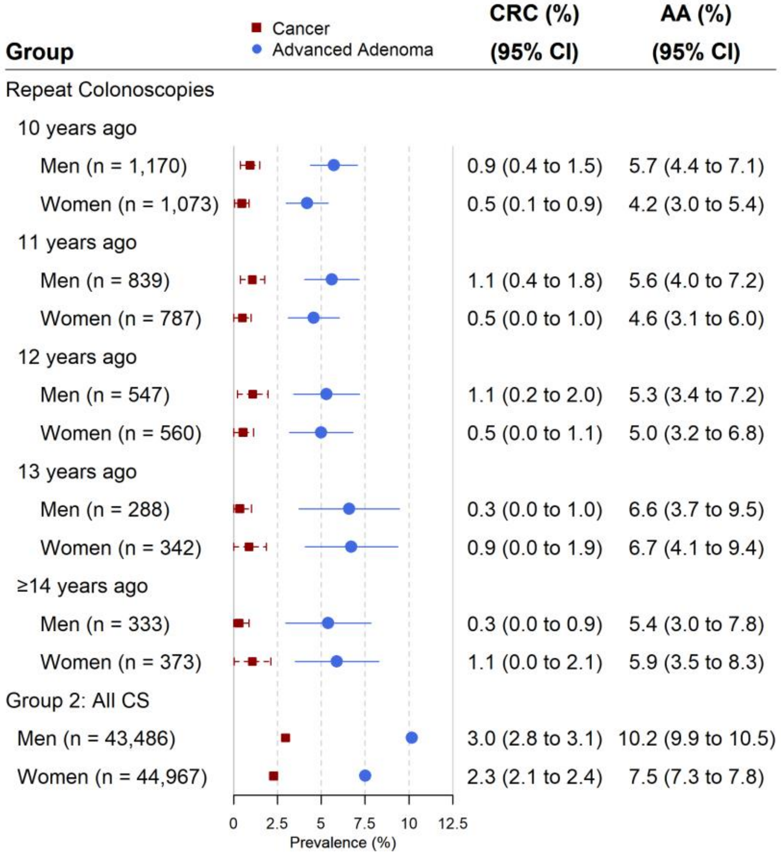


| Group | CRC (%) (95% CI) | AA (%) (95% CI) |
|---|---|---|
| Repeat Colonoscopies | | |
| 10 years ago | | |
| Men (n = 1,170) | 0.9 (0.4 to 1.5) | 5.7 (4.4 to 7.1) |
| Women (n = 1,073) | 0.5 (0.1 to 0.9) | 4.2 (3.0 to 5.4) |
| 11 years ago | | |
| Men (n = 839) | 1.1 (0.4 to 1.8) | 5.6 (4.0 to 7.2) |
| Women (n = 787) | 0.5 (0.0 to 1.0) | 4.6 (3.1 to 6.0) |
| 12 years ago | | |
| Men (n = 547) | 1.1 (0.2 to 2.0) | 5.3 (3.4 to 7.2) |
| Women (n = 560) | 0.5 (0.0 to 1.1) | 5.0 (3.2 to 6.8) |
| 13 years ago | | |
| Men (n = 288) | 0.3 (0.0 to 1.0) | 6.6 (3.7 to 9.5) |
| Women (n = 342) | 0.9 (0.0 to 1.9) | 6.7 (4.1 to 9.4) |
| ≥14 years ago | | |
| Men (n = 333) | 0.3 (0.0 to 0.9) | 5.4 (3.0 to 7.8) |
| Women (n = 373) | 1.1 (0.0 to 2.1) | 5.9 (3.5 to 8.3) |
| Group 2: All CS | | |
| Men (n = 43,486) | 3.0 (2.8 to 3.1) | 10.2 (9.9 to 10.5) |
| Women (n = 44,967) | 2.3 (2.1 to 2.4) | 7.5 (7.3 to 7.8) |

**eFigure (continued). Findings at Screening Colonoscopy in 2013-2019, in Repeat Screening Participants and Overall, stratified by Age, Sex and Intervals**

ADN, prevalence of any advanced neoplasm; ANN, prevalence of any neoplasm; CI, confidence interval.

© 2023 Heisser T et al. *JAMA Internal Medicine.*
