# Supplementary material for: Prevalence of Colorectal Neoplasia 10 or More Years After a Negative Screening Colonoscopy in 120 000 Repeated Screening Colonoscopies
Source: JAMA Intern Med. 2023 Jan 17;183(3):183–90. doi: 10.1001/jamainternmed.2022.6215 (PMC9857826; doi:10.1001/jamainternmed.2022.6215)
Supplement: Supplement 2. — Data sharing statement [file jamainternmed-e226215-s002.pdf]

Data Sharing Statement

Heisser. Prevalence of Colorectal Neoplasia 10 or More Years After a Negative Screening Colonoscopy. *JAMA Intern Med*. Published January 17, 2023.
doi:10.1001/jamainternmed.2022.6215

Data
Data available: No
